# *Francisella tularensis*, Tularemia and Serological Diagnosis

**DOI:** 10.3389/fcimb.2020.512090

**Published:** 2020-10-26

**Authors:** Max Maurin

**Affiliations:** ^1^ Centre National de Référence Francisella tularensis, Laboratoire de Bactériologie, Institut de Biologie et de Pathologie, Centre Hospitalier Universitaire Grenoble Alpes, Grenoble, France; ^2^ Laboratoire Techniques de l’Ingénierie Médicale et de la Complexité Informatique—Mathématiques et Applications (TIMC-IMAG), Université Grenoble Alpes, Centre National de la Recherche Scientifique (CNRS), Grenoble, France

**Keywords:** *Francisella tularensis*, tularemia, diagnosis, serological diagnosis, serological methods

## Abstract

Tularemia is a zoonotic disease caused by the bacterium *Francisella tularensis*. The predominant sources, routes of infection, and clinical manifestations of human infections greatly vary according to the geographic area considered. Moreover, clinical suspicion of tularemia is often tricky because of the lack of specificity of the clinical manifestations. Because *F. tularensis* isolation is tedious and detection of its DNA usually requires removal of infected tissues, serological techniques are most often used for diagnostic confirmation. However, these techniques are varied and poorly standardized. The microagglutination test (MAT), the indirect immunofluorescence assay (IFA), and ELISA tests are currently the most frequently used techniques. These home-made and commercial tests are mainly used for tularemia diagnosis but also seroprevalence studies. ELISA tests detect specific antibodies within two weeks of disease evaluation, compared to 2–3 weeks for MAT and IFA. However, more false-positive results are usually reported with ELISA. The long-term persistence of anti-*F. tularensis* antibodies in patients with past tularemia infection hampers the diagnostic specificity of all these tests. Also, cross-reacting antibodies have been described (especially with *Brucella* and *Yersinia* species), although usually at a low level. The immunoblotting technique can highlight these serological cross-reactions. Tularemia remains an underdiagnosed disease in most endemic areas, and the clinical presentations of this disease are evolving. It is necessary to improve further speed and accuracy of tularemia diagnosis, as well as the standardization of diagnostic procedures.

## Introduction

Tularemia is a zoonosis caused by two subspecies of *Francisella tularensis*, a Gram-negative facultative intracellular bacterium (Sjöstedt, 2007; Maurin and Gyuranecz, 2016). *F. tularensis* subsp. *tularensis* (type A) is mainly restricted to North America, although a few strains have been isolated in Slovakia and Austria (Gurycová, 1998). *F. tularensis* subsp. *holarctica* (type B) is spread throughout the Northern hemisphere but has recently been isolated in Australia (Aravena-Román et al., 2015). Modern classification schemes based on whole-genome sequencing have defined clades and sub-clades (Vogler et al., 2009). Type A strains include four main clades: A1a and A1b (mainly in the central and eastern USA), and A2a and A2b (mainly in the western USA). Type B strains include four main clades: B.4 (mainly North America, but also Scandinavia), B.6 (Western Europe and North America), B.12 (Eastern Europe and Asia), and B.16 (mainly Japan, but also in Turkey, China, and Australia). *F. tularensis* is classified as a category A potential biothreat agent by the CDC (Centers for Disease Control, USA) (Oyston et al., 2004; Maurin, 2015). A1b strains are considered the most virulent (Kugeler et al., 2009).

Human tularemia cases usually occur through contact with wild animals (especially hares and small rodents), arthropod bites (mainly *Ixodidae* ticks, and mosquitoes in Sweden and Finland), and the contaminated hydro-telluric environment (Sjöstedt, 2007; Maurin and Gyuranecz, 2016). Infection may occur through the skin (*e.g.*, contact with animals and arthropod bites), the conjunctiva (*e.g.*, finger-to-eye contamination), the oral route (*e.g.*, ingestion of contaminated food or water), or the respiratory tract (*e.g.*, inhalation of a contaminated aerosol). After a short incubation period (3–5 days on average, up to 2 weeks), patients usually suffer from flu-like symptoms. Depending on the route of infection, the disease may then evolve to one of the six classical clinical forms (Evans et al., 1985; Maurin and Gyuranecz, 2016). These include the ulceroglandular and glandular forms (regional lymphadenopathy with or without a skin inoculation lesion, respectively), the oculoglandular form (a conjunctivitis with cervical or pretragial lymphadenopathy), the oropharyngeal form (a pharyngitis with cervical lymphadenopathy), the pneumonic form (acute or subacute pneumonia), and the typhoidal form (severe sepsis with confusion). Many complications may occur in tularemia patients, the most common being lymph node suppuration (up to 30% of patients with regional lymphadenopathy), and less frequently meningitis and meningoencephalitis, heart infection, bone, and soft tissue infections, *etc.* (Evans et al., 1985; Khoury et al., 2005; Ata et al., 2013; Maurin and Gyuranecz, 2016; Rawal et al., 2017). The global death rate of tularemia is currently low (<1% in Eurasia, 2–3% in North America), but it may be much higher when A1b strains are involved (24% in (Kugeler et al., 2009)).

Tularemia diagnosis is often delayed due to late consultation of patients who suffer from mild symptoms and late clinical suspicion of tularemia by physicians because of poor specificity of clinical symptoms (Pérez-Castrillón et al., 2001; Tärnvik and Berglund, 2003; Maurin et al., 2011; Gozel et al., 2014). The isolation of *F. tularensis* from clinical samples is tedious and usually obtained in less than 10% of patients (Helvaci et al., 2000; Pérez-Castrillón et al., 2001; Tärnvik and Chu, 2007; Hepburn and Simpson, 2008; Larssen et al., 2011; Maurin et al., 2011). The bacterium may be isolated from blood cultures in patients with *F. tularensis* bacteremia or less frequently from other clinical samples, including skin ulcers, conjunctival or pharyngeal exudates, lymph node biopsies or suppurations, sputum samples, and cerebrospinal fluid. PCR-based methods are useful in localized forms of tularemia when exudates or tissue samples can be obtained. PCR tests may allow confirmation of tularemia at an early stage of the disease (*e.g.*, acute pneumonia, oculoglandular or oropharyngeal forms) or late diagnostic confirmation (*e.g.*, by testing surgically-removed suppurated lymph nodes) (Tärnvik and Chu, 2007; Hepburn and Simpson, 2008; Maurin and Gyuranecz, 2016). Due to limitations of the culture and PCR methods, tularemia diagnosis primarily relies on serological tests (Tärnvik and Chu, 2007; Hepburn and Simpson, 2008; Maurin and Gyuranecz, 2016). The purpose of the present review is to summarize the literature concerning the development and use of tularemia serological methods and to point out the shortcomings and possible improvements in this field.

## Serological Methods for the Detection of Anti-*F. tularensis* Antibodies

The methods developed for the detection and quantification of anti-*F. tularensis* antibodies are described in **Table 1**, including the *F. tularensis* strain and antigen used. Reported sensitivities and specificities of these tests are summarized in **Table 2**.

**Table 1:** Homemade methods used for titration antibodies directed against *F. tularensis* antigens.

Method	F. tularensis strain	Antigen	References
**Homemade tests**			
TAT	LVS (BB IND 157)	formalin-inactivated bacteria	(Koskela and Salminen, 1985)
MAT	Ootake tick strain	formalin-inactivated and safranine-stained bacteria	(Sato et al., 1990)
	LVS (ATCC 29648)	methanol-fixed bacteria	(Porsch-Ozcürümez et al., 2004)
	LVS (BB IND 157)	formalin-inactivated bacteria	(Syrjälä et al., 1986)
	hare type B strain	formalin-inactivated and safranine-stained bacteria	(Bevanger et al., 1988)
	human type B strain	NA	(Helvaci et al., 2000)
	LVS	formalin-inactivated and safranine-stained bacteria	(Splettstoesser et al., 2010)
	LVS	formalin-inactivated and safranine-stained bacteria	(Kiliç et al., 2012)
	LVS	formalin-inactivated bacteria	(Chaignat et al., 2014)
	LVS (NCTC 10857)	formalin-inactivated bacteria	(Yanes et al., 2018)
LAT	Ft	formalin-inactivated and sonicated bacteria	(Rastawicki et al., 2015)
IFA	LVS (ATCC 29648)	methanol-fixed bacteria	(Porsch-Ozcürümez et al., 2004)
	human type B strain	formalin-inactivated bacteria	(Maurin et al., 2011)
ELISA	LVS	Lipopolysaccharide extract	(Carlsson et al., 1979)
	NA	formalin-inactivated and sonicated bacteria	(Viljanen et al., 1983)
	LVS (BB IND 157)	formalin-inactivated and sonicated bacteria	(Koskela and Salminen, 1985)
	LVS (BB IND 157)	Bacterial sonicate	(Syrjälä et al., 1986)
	LVS (ATCC 29648)	Lipopolysaccharide extract	(Porsch-Ozcürümez et al., 2004; Schmitt et al., 2005)
	LVS (ATCC29684)	Lipopolysaccharide extract	(Chaignat et al., 2014)
CF-ELISA	LVS (BB IND 157)	formalin-inactivated and sonicated bacteria	(Koskela and Salminen, 1985)
cELISA	hare type B strain (NVF1)	Lipopolysaccharide extract	(Sharma et al., 2013)
ICT	NA	Lipopolysaccharide extract (Micromun ^®^)	(Splettstoesser et al., 2010)

TAT, tube agglutination test; MAT, microagglutination tests; LAT, latex agglutination test; IFA, immunofluorescence assay; ELISA, enzyme-linked immunosorbent assay; CF-ELISA, complement-fixing ELISA; cELISA, competitive ELISA; ICT, immunochromatographic assay; LVS, live vaccine strain.

**Table 2:** Reported overall sensitivities and specificities for homemade and commercial tularemia serological tests.

Method	References	Number of patients/controls (sera)	Detected antibodies	Cutoff titers	Sensitivity	Specificity	Tularemia case definition or gold standard
TAT	(Koskela and Salminen, 1985)	50 (141)/none	mainly IgM	≥10	85.1%	NA	Clinically typical + positive serology
				≥80	67.4%	NA	
MAT	(Viljanen et al., 1983)	70 (70)/none	mainly IgM	≥80	95.7%	NA	≥4fold rise in MAT or ELISA titers
	(Helvaci et al., 2000)	205 (205)/none	mainly IgM	≥80	94.1%	NA	CCF + positive culture (10) or MAT ≥ 1:20
				≥160	85%	NA	
	(Porsch-Ozcürümez et al., 2004)	50 (50)/50 (50)	mainly IgM	>16	100%	100%	CCF + seroconversion
				≥64	92%	100%	
	(Maurin et al., 2011)	93 (129)/281 (287)	mainly IgM	≥160	50.5%	99.3%	CCF + positive culture or PCR, or MAT or IFA ≥ 160, or seroconversion or ≥4fold rise in antibody titers
	(Kiliç et al., 2012)	42 culture-confirmed	mainly IgM	≥160	97.6%		Positive culture
	(Sharma et al., 2013)	19 (34)/50 (50)	mainly IgM	≥10	82.4%	100%	≥4fold rise in TAT or MAT titers
	(Yanes et al., 2018)	74 (122)/134 (134)	mainly IgM	≥80	75.3%	98.5%	CCF + positive culture or PCR, or seroconversion, or ≥4fold rise in antibody titers
LAT	(Rastawicki et al., 2015)	77/309	mainly IgM	≥25	98.7%	100%	TAT and ELISA
IFA	(Porsch-Ozcürümez et al., 2004)	50 (50)/50 (50)	all	>80	100%	92%	same as MAT
				>320	94%	100%	
	(Maurin et al., 2011)	93 (129)/281 (287)	IgM	≥160	49.4%	99.3%	same as MAT
			IgG	≥160	77.4%	98.6%	
	(Yanes et al., 2018)	74 (122)/134 (134)	IgM	≥80	72.5%	99.3%	same as MAT
			IgG	≥160	74.5%	99.3%	
ELISA	(Carlsson et al., 1979)	28 (28)/48 (48)	all	≥500 units	96.4%	97.9%	*F. tularensis* detection by culture or immunofluorescence
	(Viljanen et al., 1983)	70 (70, first two weeks of evolution) /none	IgM	OD ≥ mean + 2SD of controls	21.4%	NA	same as MAT
			IgA		28.6%	NA	
			IgG		35.7%	NA	
			at least one		43%	NA	
	(Koskela and Salminen, 1985)	50 (91)/none	IgM	≥100 units	83.5%	NA	same as TAT
			IgA		80.2%	NA	
			IgG		87.9%	NA	
	(Bevanger et al., 1988)	44 (44)/50 (50)	IgM	OD ≥ mean+ 3SD of controls	97.5%	100%	CCF + MAT ≥ 80
			IgA		97.5%	100%	
			IgG		97.5%	100%	
	(Porsch-Ozcürümez et al., 2004)	50 (50)/50 (50)	all	OD > 0.648	100%	98%	same as MAT
				OD > 0.780	98%	100%	
	(Schmitt et al., 2005)	75 (75)/1,149 (1,149)	IgM	OD > mean+ 3SD of controls	89.3%	99.5%	Clinically evident tularemia cases
			IgA		96%	98.9%	
			IgG		85.3%	98.2%	
			at least one		99%	97.1%	
	(Sharma et al., 2013)	19 (34)/50 (50)	all	OD ≥ 0.61	94.1%	98%	same as MAT
	(Chaignat et al., 2014)	110 (135)/168 (168)	all	OD ≥ 0.18	95.6%	76.6%	Positive MAT
CF-ELISA	(Koskela and Salminen, 1985)	50 (91)/none	all	≥100 units	81.9%	NA	same as TAT
cELISA	(Sharma et al., 2013)	19 (34)/50 (50)	all	8.5% rate of inhibition	97.1%	100%	same as MAT
Serazym ^®^ ELISA	(Chaignat et al., 2014)	110 (135)/168 (168)	all	(M)	97%	91.5%	same as ELISA
Serion ^®^ ELISA	(Chaignat et al., 2014)	110 (135)/168 (168)	IgM	OD ≥ 0.449 (M)	94.8%	96.8%	same as ELISA
			IgG	OD ≥ 0.619 (M)	96.3%	96.8%	
	(Yanes et al., 2018)	74 (122)/134 (134)	IgM	OD ≥ 0.449 (M)	88.2%	94.8%	same as MAT
			IgG	OD ≥ 0.619 (M)	86.3%	95.5%	
			IgM	OD ≥ 0.9	86.3%	97.8%	
			IgG	OD ≥ 1.4	84.3%	97.8%	
ICT	(Splettstoesser et al., 2010)	50 (58)/58 (58)	IgG mainly	Visual	98.3%	96.6%	Positive MAT
Virapid ^®^ ICT	(Kiliç et al., 2012)	106 (203)/236 (236)	all	≥0.5 visual Intensity	99.3%	94.6%	same as MAT
		42 culture-confirmed			97.6%		same as MAT
	(Chaignat et al., 2014)	110 (135)/168 (168)			97%	84%	same as ELISA
	(Yanes et al., 2018)	74 (122)/134 (134)			90%	83.6%	same as MAT

TAT, tube agglutination test; MAT, microagglutination tests; LAT, latex agglutination test; IFA, immunofluorescence assay; ELISA, enzyme-linked immunosorbent assay; CF-ELISA, complement-fixing ELISA; cELISA, competitive ELISA; ICT, immunochromatographic assay; CCF, compatible clinical findings; (M), manufacturer cutoff titer.

### Agglutination Tests

#### Literature Data

The agglutination reaction of *F. tularensis* was first reported by Francis and Evans in 1926 (Francis and Evans, 1926) using the tube agglutination test (TAT). This test was used for decades for tularemia diagnosis (Ransmeier and Ewing, 1941) and then replaced by the microagglutination test (MAT), which was considered more rapid (1–2 h *versus* one day), more comfortable to perform and interpret, and more sensitive in the early stage of the disease (Massey and Mangiafico, 1974; Brown et al., 1980). Also, the MAT uses much less amount of antigen than the TAT. Both tests primarily detect IgM-type antibodies although IgG antibodies also contribute to the agglutination.

Koskela and Salminen (1985) evaluated a MAT test using 123 sera collected from 50 recent tularemia cases occurring in Finland between 1967 and 1978. Most patients were infected through mosquito bites and developed the ulceroglandular (73%) or glandular (25%) forms of the disease. For 114 sera collected during the first 30 days of disease onset, sensitivities of 81.6 and 59.6% were found for cutoff titers of ≥10 or ≥80, respectively. Most sera collected during the first 11 days were MAT negative, and thus sensitivities were 97.6 and 72.6% when considering only serum samples collected between 11 and 30 days of disease evolution.

Bevanger et al. (1988) tested acute-phase sera from 44 tularemia patients infected in 1984–1985 during an outbreak in central Norway. Confirmed tularemia cases had compatible clinical findings and a previous MAT titer ≥80. A sensitivity of 91% was found for a cutoff titer of ≥80. Sera from 50 blood donors had MAT titers <20 (100% specificity).

Sato et al. (1990) evaluated sera with TAT titers between 20 and 640 collected from ten tularemia patients. The MAT detected serum agglutinins nine days earlier and at titers 8–64 times higher compared to the TAT.

Helvaci et al. (2000) evaluated 205 sporadic or outbreak cases of tularemia occurring in the Bursa region (Anatolia, Turkey) between 1988 and 1998. Tularemia case definition included compatible clinical symptoms and a positive *F. tularensis* culture (10 cases) or a MAT titer ≥20 (195 cases). Their MAT test gave titers ≥160 for 85% of patients, and ≥80 for 94.1%. Interestingly, half of the patients with a positive *F. tularensis* culture had a negative MAT.

Porsch-Ozcürümez et al. (2004) evaluated 50 sera from Sweden tularemia cases (confirmed by seroconversion) and 50 healthy blood donors. The sensitivity and specificity of their MAT at a cutoff titer of >16 were 100%. MAT titers were ≥64 in 46/50 (92%) tularemia patients, but <16 in all healthy controls.

We evaluated by MAT (using a human type B strain as an antigen) 129 sera from 93 tularemia patients, and 287 sera from 281 controls (Maurin et al., 2011). We defined tularemia cases as the combination of compatible clinical findings with either a positive *F. tularensis* culture or PCR, a positive serology (MAT or IFA, cutoff titer ≥160), seroconversion or a fourfold rise in antibody titers. We found a diagnostic sensitivity of 50.5% and specificity of 99.3%. We later used a second MAT (with the LVS strain as an antigen) to test 122 sera from 74 tularemia patients infected in France between 2006 and 2015, and 134 sera from controls (Yanes et al., 2018). Using a cutoff titer of ≥80, we found a sensitivity of 75.3% and a specificity of 98.5%. In both cases, the MAT was evaluated in a real clinical situation, with many sera taken during the early phase of tularemia.

More recently, Rastawicki et al. (2015) developed a latex agglutination test (LAT). LAT titers corresponded to the serum dilution allowing agglutination of at least 50% of the latex particles, with a cutoff ≥25. Among 77 tularemia patients (defined by positive TAT or ELISA tests), 76 (98.7%) were positive by LAT. The LAT was negative in 36 sera from suspected tularemia cases but negative TAT or ELISA tests, 134 sera from blood donors, and 139 sera from patients with other bacterial infections. As compared to routine diagnostic tests, including a TAT, a homemade ELISA, and the Serion ^®^ ELISA classic *Francisella tularensis* (referred hereafter as the Serion ^®^ ELISA, Institut Virion/Serion GmbH, Würzburg, Germany), the LAT displayed a sensitivity of 97.4–100% and a specificity of 100%.

#### Comments

Because it is widespread and straightforward, the MAT is still considered a reference serological method (World Health Organization, 2007). This test has often been used as a gold standard to evaluate the performances of newly developed serological methods. However, literature data indicate that the MAT shares the same limitations as other serological tests for tularemia diagnosis. Studies evaluating the actual clinical performances of MATs for tularemia diagnosis are scarce. In most cases, such performances were evaluated in ill-defined tularemia cases and control subjects. For example, tularemia cases were often defined by compatible clinical findings and a previously positive MAT test (Bevanger et al., 1988; Helvaci et al., 2000; Porsch-Ozcürümez et al., 2004). Likewise, control patients were often healthy blood donors (Bevanger et al., 1988; Porsch-Ozcürümez et al., 2004). Thus, the high sensitivities and specificities reported for MATs may not reflect their actual performances in a real clinical setting. Using multi-criteria definitions for tularemia and control cases, in a real clinical situation, we found lower sensitivities of our MAT tests (*i.e.*, 50.5 and 75.3%) when cutoff titers (≥80 and ≥160, respectively) were defined to obtained specificities higher than 98% (Maurin et al., 2011; Yanes et al., 2018). Indeed, a crucial point for the MAT is the choice of the cutoff titer. High sensitivities (82.4–100%) were reported for cutoff titers of 10–20 (Koskela and Salminen, 1985; Porsch-Ozcürümez et al., 2004). In contrast, lower and more variable sensitivities (50–97.6%) were reported for cutoff titers of 80–160 (Bevanger et al., 1988; Helvaci et al., 2000). False-positive results due to antigenic cross-reaction are much more frequent for agglutination titers in the range of 10–40 (Syrjälä et al., 1986). Protocols described in the WHO guidelines propose cutoff titers of ≥128 for the MAT and ≥160 for the TAT (World Health Organization, 2007).

### Immunofluorescence

#### Literature Data

Porsch-Ozcürümez et al. (2004) tested sera from 50 Swedish tularemia cases and 50 healthy blood donors using an indirect immunofluorescence assay (IFA). The sensitivities and specificities were respectively 100 and 92% at a cutoff titer of >160, and 94 and 100% at a cutoff titer of >320. However, they considered the IFA as poorly reliable because of difficulties and subjectivity in determining the endpoint titers.

We first developed an IFA using a human type B strain of *F. tularensis* as an antigen (Maurin et al., 2011). We tested 93 tularemia patients (129 sera) and 281 controls (287 sera). We used a multiparametric definition of tularemia cases (see *MAT* section). At a cutoff titer of ≥160, the IFA displayed sensitivities and specificities of 49.4 and 99.3% for IgM and 77.4 and 98.6% for IgG. All sera collected during the first ten days of disease evolution were negative. We later used a second IFA (with the LVS strain as an antigen) to test sera from 74 tularemia patients (122 sera) and 134 controls (Yanes et al., 2018). Sensitivities and specificities were 72.5 and 99.3% for IgM (cutoff ≥80), and 74.5 and 99.3% for IgG (cutoff ≥160).

#### Comments

As compared to agglutination tests, the IFA allows the titration of different immunoglobulin classes, usually IgM and IgG. A well-known drawback of this technique is the subjective reading of fluorescence, which may lead to operator-related variations in the determined antibody titers. However, more accurate antibody titer determination can be obtained by considering as the antibody titer the reciprocal of the highest serum dilution that still allows easy reading of fluorescence. Despite these technical difficulties, literature data indicate that the IFA method provides comparable results to the MAT tests. As for the MAT, the sensitivity of the IFA may be low in real clinical situations because of a lack of detection of antibodies in sera collected during the first two weeks following disease onset (Maurin et al., 2011; Yanes et al., 2018).

### Enzyme-Linked Immunosorbent Assay

#### Literature Data

Using a homemade ELISA, Carlsson et al. (1979) tested serum samples from 28 recent tularemia cases (3–5 weeks of evolution) and 48 healthy volunteers. Tularemia diagnosis was confirmed by culture (three cases) or immunofluorescence detection of *F. tularensis* in clinical samples. For tularemia patients, relative ELISA titers (both IgM and IgG) were ≥2000 for 23 (82.1%) cases, between 500 and 2000 for four cases, and <500 for one case. Almost all healthy controls (47/48) had titers <500, while the remaining had a titer of 1,400. A relative ELISA titer ≥500 was considered positive with a sensitivity of 96.4 and a specificity of 97.9%.

Viljanen et al. (1983) developed an ELISA using a bacterial sonicate (S-ELISA). Specific IgM, IgA, and IgG antibodies were determined in paired sera from 106 patients with suspected tularemia. A fourfold or higher rise in antibody titers was found in 70 patients by MAT (thus considered proven tularemia cases) but 67 patients by S-ELISA. When considering only the first serum sample of the 70 MAT-positive patients, 30 (43%) and 12 (17%) patients were positive with the S-ELISA (at least one immunoglobulin type) and MAT, respectively. Thus, the S-ELISA detected specific antibodies earlier than the MAT.

Koskela and Salminen (1985) evaluated an ELISA and a complement-fixing ELISA (CF-ELISA) tests in 50 Finland patients with the ulceroglandular (73%) or glandular (25%) forms of tularemia. When testing sera collected between 0 and 40 days following disease onset, sensitivities (cutoff ≥100 units) were 83.5, 80.2, and 87.9% for the ELISA (IgM, IgA, and IgG, respectively) and 81.9% for CF-ELISA. Most patients did not develop antibodies during the first five days of disease evolution. Thus, when considering serum samples collected between 6 and 40 days, the sensitivities of the same tests were 92.5, 90, 95, and 91.7%.

Syrjälä et al. (1986) tested 141 sera from 76 tularemia patients (36 pneumonic, 32 ulceroglandular and eight oropharyngeal forms) infected in Northern Finland in 1982–1983, using homemade MAT (cutoff ≥80) and ELISA (cutoff ≥100) tests. Both methods detected significant antibody titers among the 52 serum samples collected 3–4 weeks after disease onset. In contrast, during the first two weeks of disease evolution, five serum samples were positive by ELISA but negative with MAT.

Bevanger et al. (1988) tested sera from 44 outbreak tularemia cases and 50 blood donors. They considered patients with compatible clinical findings and a MAT titer ≥80 as confirmed tularemia cases. Serum samples with ELISA units ≥mean + 3 SD of the control group were considered positive. ELISA detected significant IgM, IgA, or IgG antibodies in 43 (97.5%) of the patients and the three immunoglobulin types in 41 (93%). None of the controls displayed significant ELISA titers.

Porsch-Ozcürümez et al. (2004) tested by ELISA sera from 50 tularemia patients with MAT seroconversion and 50 healthy blood donors. The ELISA displayed sensitivities and specificities of 100 and 98% at OD >0.648, and 98 and 100% at OD >0.780. Using the same ELISA tests, the same team (Schmitt et al., 2005) evaluated sera from 75 clinically evident tularemia cases and 1149 healthy young adults as controls. They reported sensitivities of 96, 89.3, 85.3, and 98.7% for IgA, IgM, IgG, and the combination of these three immunoglobulin types, respectively. Specificities were 98.9, 99.5, and 98.2% for IgA, IgM and IgG, respectively.

Sharma et al. (2013) developed a competitive ELISA (cELISA) test based on the ability of serum antibodies to inhibit the binding of monoclonal antibodies to *F. tularensis* lipopolysaccharide antigen. The main advantage of this test is the possibility to titrate anti-*F. tularensis* antibodies in humans and different animal species with the same method and at the same time, with no need to use a species-specific conjugated secondary antibody. They tested 34 sera from 19 tularemia patients (confirmed by a significant rise in TAT or MAT titers) and 50 sera from healthy individuals. The cELISA displayed a sensitivity of 97.1% and a specificity of 100%, comparable to those of two homemade tests, ELISA (94.1 and 98%) and MAT (82.4 and 100% at a cutoff ≥10).

Chaignat et al. (2014) recently evaluated two commercially available ELISA assays: the Serazym ^®^ anti-*Francisella tularensis* ELISA (referred hereafter as the Serazym ^®^ ELISA, Seramum Diagnostica GmbH, Heidesse OT Wolzig, Germany) and the Serion ^®^ ELISA. Both tests use a *F. tularensis* LPS extract as an antigen. They compared these tests with two homemade MAT and ELISA tests. They tested 135 sera from 110 consecutive tularemia patients infected in an endemic area in Serbia between 1999 and 2009. Tularemia cases were defined by compatible clinical symptoms and at least one positive MAT test (cutoff ≥20). Controls included 94 sera from patients suffering from other diseases (including 20 culture-proven brucellosis cases in Lebanon) and 74 sera from German blood donors. As compared to MAT, the sensitivities and specificities of ELISA tests were as follows: Serion ^®^ ELISA IgM, 94.8 and 96.8%; Serion ^®^ ELISA IgG, 96.3 and 96.8%; Serazym ^®^ ELISA, 97.0% and 91.5; and 95.6 and 76.6% for the homemade ELISA test at OD ≥0.18. As compared to the MAT, detection of IgM or IgG using the Serion ^®^ ELISA was the most efficient method. Interestingly, no cross-reactions were observed with the 20 sera from brucellosis patients.

More recently, we evaluated the Serion ^®^ ELISA in comparison to homemade MAT and IFA tests (Yanes et al., 2018). We tested 122 sera from 74 French tularemia patients and 134 controls. The Serion ^®^ ELISA tests, using cutoff titers advocated by the manufacturer, found sensitivities and specificities of 88.2 and 94.8% for IgM, and 86.3 and 95.5% for IgG. These ELISA tests detected specific antibodies earlier than the MAT and IFA tests (2–3 weeks *versus* 3–4 weeks after onset of symptoms). An overall specificity close to 95% may be associated with a low positive predictive value in countries where tularemia is a rare disease. Therefore, we tested higher cutoffs to obtain a specificity close to 98% and found sensitivities of 86.3% for IgM (OD ≥ 0.9) and 84.3% for IgG (OD ≥ 1.4). The new cutoffs remarkably increased specificities without significant alteration of sensitivities, which could increase the positive predictive value of the test in tularemia low endemic areas.

#### Comments

Most ELISA tests developed for tularemia diagnosis used as antigens either a *F. tularensis* lipopolysaccharide extract (Carlsson et al., 1979; Porsch-Ozcürümez et al., 2004; Schmitt et al., 2005; Sharma et al., 2013; Chaignat et al., 2014) or a sonicate of whole bacteria (Viljanen et al., 1983; Koskela and Salminen, 1985; Syrjälä et al., 1986). As for IFA, the ELISA method can detect IgM or IgG types of antibodies separately. When considering as positive sera with significant IgM, IgA or IgG titers, sensitivities and specificities of ELISA tests varied from 96.4 to 100%, and 91.5 to 100%, respectively (Carlsson et al., 1979; Bevanger et al., 1988; Porsch-Ozcürümez et al., 2004; Schmitt et al., 2005; Sharma et al., 2013; Chaignat et al., 2014). These tests performed as well or even better than MAT tests. However, here again, sensitivities were often determined in poorly defined tularemia cases and pecificities only in healthy blood donors. Using a complement-fixing ELISA test, Koskela et al. (Koskela and Salminen, 1985) reported lower sensitivity and specificity (81.9 and 91.7%, respectively). When evaluating the Serion ^®^ ELISA (using the manufacturer’s cutoffs) in a real clinical situation, we reported sensitivities and specificities of 88.2 and 94.8% for IgM, and 86.3 and 95.5% for IgG. Therefore, although ELISA tests are useful diagnostic tools for tularemia diagnosis, their actual performances should be further evaluated in real clinical situations.

ELISA methods have several advantages compared to older methods. Several studies have shown that significant antibody titers are usually detected earlier with ELISA than with TAT, MAT, and IFA tests (Viljanen et al., 1983; Syrjälä et al., 1986; Yanes et al., 2018). ELISA methods generally make it possible to detect specific antibodies as early as the second week of the evolution of the disease. In contrast, TAT, MAT, and IFA methods usually detect antibodies only during the third week (see the specific section above). ELISA methods are also better adapted for testing a large number of serum samples, which is specifically useful in epidemic situations and for seroepidemiological surveys.

### Immunochromatographic Test

#### Literature Data

Splettstoesser et al. (2010) developed an immunochromatographic test (ICT) that primarily detected IgG antibodies directed against *F. tularensis* LPS. They tested 58 sera from 50 tularemia patients (including 3 cases confirmed by culture, 14 by PCR, two by seroconversion, and 20 by a significant change in antibody titers) and five LVS-vaccinated individuals. They also tested 58 sera from patients infected with other bacterial species as controls. Compared to a homemade MAT, the ICT displayed an overall sensitivity of 98.3% and specificity of 96.6%. The authors considered that ICT could be useful for the rapid diagnosis of tularemia in humans and animals, especially for field investigations.

Kiliç et al. (2012) evaluated the commercial VIRapid ^®^ immunochromatographic (ICT) test that uses an LPS extract from the LVS strain (referred hereafter as the VIRapid ^®^ ICT, Vircell ^®^, Granada, Spain). Both the ICT and a MAT (taken as a gold standard) were used to test 203 sera (collected 4 to 42 days after disease onset) from 106 confirmed tularemia cases (97.2% oropharyngeal forms) occurring between 2009 and 2011 in Turkey. Controls included 236 sera from 85 blood donors and 151 patients with diseases other than tularemia. For tularemia patients, 140/203 (68.9%) sera were ICT positive. As compared to the MAT, the ICT displayed an overall sensitivity of 99.3% and a specificity of 94.6%. The ICT was positive in 97.6% of the 42 culture-confirmed tularemia cases. False-positive results were mainly found with sera from brucellosis patients.

Chaignat et al. (2014) also evaluated the VIRapid ^®^ ICT against sera from 110 tularemia patients and 168 controls. As compared to MAT, they reported a sensitivity of 97% and a specificity of 84%.

In our hands (Yanes et al., 2018), the same test displayed a sensitivity of 90% and a specificity of 83.6% when testing 256 sera from 208 patients, including 74 tularemia patients and 134 controls.

#### Comments

Literature data indicate good performances of homemade or commercial ICT tests for tularemia diagnosis, with reported sensitivities of 97–99.3% and specificities of 84–96.6% taking the MAT as a reference test (Splettstoesser et al., 2010; Kiliç et al., 2012; Chaignat et al., 2014). When using a multi-criteria definition of tularemia cases (and not a positive MAT test), the ICT displayed lower sensitivities of 68.9% (Kiliç et al., 2012) and 90% (Yanes et al., 2018) sera from tularemia patients. In a later study (Yanes et al., 2018), the specificity was only 83.6%, comparable to that reported by Chaignat et al. (2014). Thus, available data indicate that ICTs may be useful as screening tests for tularemia but, because of their low specificity, positive results must be confirmed using a more specific test. Further evaluation of these tests in real clinical situations is needed.

### Immunoblots

#### Literature Data

Bevanger et al. (1988) evaluated by Western blot 12 sera from tularemia patients against *F. tularensis* outer membrane (OM) antigens. High IgG antibody response was observed against two or more OM antigens of 11, 19, 43, and 50 kDa. All 12 sera detected the 43 kDa OM although with variable intensity. The same authors (Bevanger et al., 1989) developed a competition ELISA test to evaluate the antibody response against the 43 kDa antigen in paired serum samples from 23 tularemia patients compared to 25 blood donors. When using a 1:64 dilution of sera, they found a sensitivity of 95.7% and a specificity of 96%. The authors concluded that the 43 kDa antigen could be useful for the serological diagnosis of tularemia.

Using an LPS extract from LVS cells as an antigen, Porsch-Ozcürümez et al. (2004) tested serum samples from 50 tularemia cases (previously confirmed by seroconversion) and 50 blood donors. Their Western blot technique allowed differentiation between patients and controls.

Schmitt et al. (2005) tested 75 sera from clinically evident tularemia cases and 1,149 from healthy young adults. The immunoblots revealed an LPS-specific ladder (15–98 kDa) for all tularemia cases (100% sensitivity). However, the western blot assay was positive in 36 of the 1,149 healthy controls, giving a 97% specificity.

More recently, Chaignat et al. (2014) developed a Western blot assay using *F. tularensis* LPS as an antigen. Compared to a homemade MAT test, they reported sensitivity and specificity of 93.3 and 83%, respectively.

The immunoblotting technique has also been used to determine the *F. tularensis* immunoreactive proteins using sera from infected or vaccinated persons (Havlasová et al., 2002; Eyles et al., 2007; Sundaresh et al., 2007; Nakajima et al., 2016). These studies have shown that the most immunoreactive *F. tularensis* included the chaperone proteins dnaK and groEL, two chaperonins (10 and 60 kDa), the elongation factor EF-Ts, membrane proteins (23 kDa hypothetical protein, 17 kDa TUL4 protein, OmpH and FopA), enzymes (pyruvate dehydrogenase, acetyl-CoA carboxylase, pyrrolidone-carboxylate peptidase, isocitrate dehydrogenase, glutamine synthetase, *etc*.) and conserved hypothetical lipoproteins (including that encoded by *lpnA*). Thus, although the antibody response in tularemia patients is considered mainly directed against *F. tularensis* lipopolysaccharide (Waag et al., 1992), many bacterial proteins are also immunogenic and may serve to construct new diagnostic tools.

#### Comments

The immunoblotting technique has been proposed as a confirmatory test in patients with positive MAT, IFA, or ELISA tests. The ladder-like band pattern obtained with *F. tularensis* LPS is considered as typical for this species (Porsch-Ozcürümez et al., 2004; Schmitt et al., 2005; Jenzora et al., 2008; Chaignat et al., 2014; Rossow et al., 2015; Zákutná et al., 2015; Njeru et al., 2017). However, in many studies, western blot could not confirm a large number of positive tests obtained with other methods (Porsch-Ozcürümez et al., 2004; Schmitt et al., 2005; Rossow et al., 2015; Zákutná et al., 2015; Njeru et al., 2017). This difference may reflect the higher specificity of the immunoblot technique but also low sensitivity of this method in patients with low antibody titers. Several studies have tried to dissect the immune response of patients infected with *F. tularensis* to develop more sensitive and specific serological tests (Havlasová et al., 2002; Eyles et al., 2007; Sundaresh et al., 2007; Nakajima et al., 2016). Although these studies have well defined the most immunodominant *F. tularensis* antigens, they have not given rise to the development of new serological tests with improved performances compared to those currently used for tularemia diagnosis. This observation may be partly explained by the low-level antibody response observed in infected or vaccinated persons against *F. tularensis* proteins compared to LPS.

## Antibody Response Kinetics In Naturally Infected Tularemia Patients

The sensitivities of tularemia serological methods at a different time following *F. tularensis* infection are presented in **Table 3**. These variable sensitivities reflect the kinetics of the antibody response in tularemia patients, which was evaluated using the TAT, MAT, IFA, and ELISA methods (Francis and Evans, 1926; Ransmeier and Ewing, 1941; Carlsson et al., 1979; Koskela and Salminen, 1985; Syrjälä et al., 1986; Maurin et al., 2011). Specific antibodies developed in *F. tularensis* infected patients are usually detected 2–3 weeks and peak at 4–6 weeks following symptoms onset. The antibody response has been reported to be similar in tularemia patients with different clinical forms or the severity of this disease (Syrjälä et al., 1986). Only a few patients never seroconvert or develop a very late antibody response (Syrjälä et al., 1986). Interestingly, IgM antibodies are usually detected only a few days before, at the same time or even after IgG antibodies (Koskela and Salminen, 1985; Syrjälä et al., 1986; Bevanger et al., 1989). The antibody response may also be transient (less than one month) (Syrjälä et al., 1986). Most often, antibody titers slowly decline over time, although more rapidly for IgM than for IgG antibodies. However, residual antibody titers (both IgM and IgG) may persist for years (see the specific section below). Therefore, for tularemia, the presence of IgM antibodies is not a reliable indicator of recent infection (Koskela and Salminen, 1985; Tärnvik and Berglund, 2003).

**Table 3:** Reported sensitivities for homemade and commercial tularemia serological tests according to disease evolution.

References	Method	Antibody types	Cutoff titers	Sensitivity at different time intervals of disease onset for (n) serum samples		
				**1^st^ week (19)**	**2^nd^ week (13)**	**weeks 3–5 (28)**
(Carlsson et al., 1979)	ELISA	all	≥500 relative titer	15.8%	50%	96%
				**0–5 days (11)**	**6–10 days (12)**	**11–20 days (30)**
(Koskela and Salminen, 1985)	TAT	mainly IgM	≥10	7.1%	65%	97.8%
		mainly IgM	≥80	0%	15%	71.7%
	CF-ELISA	all	≥100	18.2%	70%	88%
	ELISA	IgM	≥100	18.2%	75%	90%
		IgA		9.1%	58.3%	90%
		IgG		36.4%	91.7%	90%
		At least one		36.4%	91.7%	96.7%
				**1^st^ week (NA)**	**2^nd^ week (NA)**	
(Syrjälä et al., 1986)	MAT	mainly IgM	≥10	65%	89%	
		mainly IgM	≥80	3%	52%	
	ELISA	IgM	≥100	29%	71%	
		IgA	≥100	23%	89%	
		IgG	≥100	29%	93%	
		At least one	≥100	48%	100%	
				**1^st^ week (20)**	**2^nd^ week (86)**	**weeks 3–5 (76)**
(Kiliç et al., 2012)	Virapid ^®^ ICT	All	≥0.5 visual density	20%	67.4%	96.1%
				**1^st^ week (7)**	**2^nd^ week (6)**	**weeks 3–4 (8)**
(Yanes et al., 2018)	MAT	all	≥80	0%	33.3%	75%
	IFA	IgM	≥80	0%	33.3%	62.5%
		IgG	≥160	0%	0%	87.5%
	Serion ^®^ ELISA	IgM	OD ≥ 0.45	14.3%	66.7%	100%
			OD ≥ 0.9	14.3%	66.7%	100%
		IgG	OD ≥ 0.62	0%	66.7%	100%
			OD ≥ 1.4	0%	33.3%	100%
	Virapid ^®^ ICT	all	+ to +++	14.3%	33.3%	100%

TAT, tube agglutination test; MAT, microagglutination tests; LAT, latex agglutination test; IFA, immunofluorescence assay; ELISA, enzyme-linked immunosorbent assay; CF-ELISA, complement-fixing ELISA; ICT, immunochromatographic assay.

Carlsson et al. (1979) evaluated the antibody response kinetics in 40 tularemia patients (60 sera) with recent infection. Sera were collected during the first week (n = 19), second week (13), or third to fifth weeks following disease onset (28). Significant IgM and IgG antibody titers were found in 15.8% of the patients during the first week, 50% during the second week, and 96% during the third to fifth week period.

Koskela and Salminen (1985) evaluated the antibody kinetics response [using a TAT, ELISA, and complement-fixing ELISA (CF-ELISA) methods] in 50 tularemia cases. Test sensitivities were determined for the periods 0–5 days (n = 11 sera), 6–10 days (12), 11–20 days (30), and 21–40 days (38) following disease onset. The ELISA (cutoff ≥100 units; IgM, IgA, and IgG combined) displayed sensitivities of 36.4, 91.7, 96.7, and 100% for the four periods studied, respectively. The CF-ELISA (cutoff ≥ 100 units) displayed sensitivities of 18.2, 70, 88, and 100%. The TAT had sensitivities of 0, 15, 71.7, and 96% for a cutoff titer ≥80. Tus, the ELISA methods performed better for early detection of specific antibodies.

Syrjälä et al. (1986) tested 141 sera from 76 Finland tularemia patients. The MAT (cutoff ≥ 80) and ELISA (cutoff ≥ 100) were positive for all 52 sera collected 3–4 weeks after disease onset. For sera collected during the first week of illness, sensitivities were 48% for the ELISA (29% for IgM, 23% for IgA, and 29% for IgG), and 65 and 3% for the MAT (cutoff >or=10 and >or=80, respectively). During the second week, all sera were ELISA positive for at least one immunoglobulin type (71% for IgM, 89% for IgA, and 93% for IgG), whereas 89 and 52% were MAT positive (cutoffs ≥ 10 and ≥80, respectively).

Kiliç et al. (2012) evaluated the VIRapid ^®^ ICT in 203 sera from 106 tularemia cases. The ICT was positive in 20% of patients after one week of disease evolution, 67.4% after the second week, and 96.1% after the third week.

We evaluated the antibody response kinetics in 74 French tularemia patients, using homemade MAT and IFA tests, and the commercial Serion ^®^ ELISA and VIRapid ^®^ ICT (Yanes et al., 2018). Cutoff titers were ≥80 for the MAT and IFA-IgM, ≥160 for IFA-IgG, and those recommended by the manufacturers for the commercialized tests. For the first week of disease evolution, we found no antibodies by the MAT, IFA, and Serion ^®^ ELISA IgG^®^ tests, but 14.3% sensitivity for both the Serion ^®^ ELISA IgM^®^, and the Virapid^®^ ICT. During the second week of illness, 33.3% of sera were positive by MAT, IFA-IgM, and Virapid^®^ ICT, and 66.7% by the Serion ^®^ ELISA for both IgM and IgG. No IgG antibodies were found using the IFA-IgG test. During the third and fourth weeks of disease evolution, sensitivities increase to 75% for the MAT, 62.5 and 87.5% for the IFA-IgM and IFA-IgG tests, respectively, and 100% for the Serion ^®^ ELISA (both IgM and IgG) and the Virapid ^®^ ICT. Thus, the study indicated that commercial tests were more sensitive in the early stage of tularemia. These high sensitivities, however, were associated with lower specificities.

Some authors have argued that early detection of low titers of specific antibodies using ELISA may not be clinically useful because of difficulties in differentiating recent infection from residual antibodies of past infection (Syrjälä et al., 1986). Nevertheless, in a clinical and epidemiological context compatible with tularemia, early detection of anti-*F. tularensis* antibodies reinforces clinical suspicion and helps to avoid diagnostic wandering.

## Antibody Response in *f. Tularensis*-vaccinated Persons

Carlsson et al. (1979) tested the humoral response of 46 persons vaccinated with the LVS strain of *F. tularensis.* Serum samples were collected before and five weeks after vaccination and tested using an ELISA method (with a phenol-water extract from the LVS strain as an antigen). Among vaccinated persons, 44 (95.6%) displayed a twofold or higher increase in serum antibody titers. However, only 23/44 (52.3%) displayed relative ELISA titers ≥500 (range 500–2000), whereas 23/28 (82.1%) patients with natural tularemia infection had titers ≥2,000. Thus, the humoral antibody response to *F. tularensis* vaccination was remarkably lower than that observed in naturally infected patients.

In 1982, Koskela and Herva, (1982a) described the immune response in 13 subjects after vaccination with the LVS strain. When using the agglutination method, titers ≥80 were detected four weeks after vaccination and picked between 4 and 8 weeks. The ELISA detected IgM antibody titers 1.8 months after vaccination, one week, and one month earlier than IgA and IgG, respectively. These specific antibodies persisted for more than one year (several years in some cases), IgG antibodies being predominant. However, vaccinated persons were not adequately protected against *F. tularensis* infection.

Waag et al. (1992) evaluated the immune response to the LVS strain (blue colony variant) in nine U.S. Army volunteers. Using an ELISA test and irradiation-killed LVS as an antigen, a twofold or higher rise in IgM, IgA, and IgG antibody titers was observed 28 days after vaccination. Fourfold or higher rise in titers was observed 56 days following vaccination in most persons. Immunoblots were positive with a specific LPS banding pattern in 5/9 tested persons from 28 days after vaccination. In a further study, the same authors (Waag et al., 1995) evaluated the immune response of 22 LVS-vaccinated persons using an ELISA method and different antigens: irradiation killed LVS cells; LVS aqueous ether extract (EEx); LVS lipopolysaccharide (LPS); and the virulent SCHU4 strain. A significant rise in IgA, IgM, and IgG antibody titers was observed with all antigens, except an IgG response against LPS. Since 80% of patients developed antibodies against EEx 14 days after vaccination, the authors advocated the use of this antigen to evaluate the immune response to *F. tularensis*, both in vaccinated and infected individuals.

## Residual anti-*f. Tularensis* Antibodies

Carlsson et al. (1979) tested serum samples from 19 patients with past tularemia infection. Residual ELISA antibody titers (relative antibody titers of 170–3,570 for IgM and 360–8,320 for IgG) were found in sera collected 2.5 years after infection. There was a clear overlap with relative antibody titers found in patients with recent tularemia infection (130–6,750 for IgM and 200–6,630 for IgG, 3–5 weeks after disease onset). However, IgG type antibodies predominated 2.5 years after infection, with an average IgG/IgM ratio of 2.6.

Koskela and Salminen (1985) evaluated the presence of residual antibodies in 23 tularemia patients seven months to 11 years following infection. TAT, ELISA, and complement-fixing ELISA (CF-ELISA) were used for antibody titration. Compared to 123 sera collected in tularemia patients 0 to 6 months following disease onset, the authors reported a marked decline in antibody titers over time, although more marked for IgM and IgA compared to IgG antibodies. However, titers above the cutoff (≥100) were found with ELISA and CF-ELISA in all sera collected seven months to 11 years following infection. TAT titers were ≥10 for 100% of sera and ≥80 for 95.6%.

Sato et al. (1990) reported that residual antibodies could be detected by MAT (Ootake tick strain of *F. tularensis* as an antigen) up to 20 years after recovery from infection.

Bevanger et al. (1994) evaluated the antibody response in 22 outbreak tularemia patients in Norway. Serum samples were collected during the acute phase of the disease (n = 22) and eight years later (n = 22). MAT antibody titers ≥40 were found in 21/22 (95.4%) acute-phase sera and 14/22 (63.5%) late sera. ELISA tests were positive in 91% of sera for IgM and 100% for IgA and IgG in the acute phase period. They were positive in 27% of sera for IgM, 55% for IgA, and 95% for IgG eight years later. This study clearly showed that anti-*F. tularensis* antibodies may persist for years after infection, but more remarkably for IgG. Thus, the authors emphasized that an IgG/IgM ratio ≥5 was suggestive of past infection. In contrast, Western blot displayed similar banding patterns for the acute and late sera.

Altogether, the long-term persistence of anti-*F. tularensis* antibodies after infection or vaccination have been reported in several studies (Francis and Evans, 1926; Ransmeier and Ewing, 1941; Carlsson et al., 1979; Koskela and Salminen, 1985; Sato et al., 1990; Bevanger et al., 1994). Residual antibodies may be responsible for serological false-positive results because titers are often above cutoffs. Thus, tularemia diagnosis can be considered confirmed only when a seroconversion or a significant (fourfold or higher) rise in antibody titers is observed between acute and convalescence phase sera.

## Serological cross-reactions between *f. Tularensis* and other Microorganisms

Carlsson et al. (1979) developed TAT and ELISA assays using as antigens an LPS extract from *F. tularensis* LVS, *B. abortus* strain 544, or *Y. enterocolitica* O:3 strain 482. Cutoff titers were ≥160 and ≥500 for the TAT and ELISA tests, respectively. Sera from 28 tularemia patients collected 3–5 weeks after disease onset were evaluated. Tularemia ELISA titers were ≥2,000 in 23 patients, 500–2,000 in four patients, and <500 in one patient. MAT titers were ≥320 in 22 patients, 160 in four patients, and <40 in two patients. Only four and three tularemia patients had significant antibody titers against *B. abortus* and *Y. enterocolitica* O:3, respectively. Crossed antibody titers against these two pathogens (500–2,000) were much lower than anti-*F. tularensis* antibody titers.

Behan and Klein (1982) tested sera from 128 tularemia and 34 brucellosis patients using specific MAT tests. All sera displayed MAT titers ≥160 (considered as the cutoff) against the homologous antigen. *B. abortus* MAT titers ≥20 were found in 48/128 tularemia serum samples. *F. tularensis* MAT titers ≥20 were found in 8/34 brucellosis serum samples. For 42 tularemia patients with homologous MAT titers between 160 and 10,240, *B. abortus* MAT titers were in the range 20–80 in 35 patients, 160–640 in five, and >640 in two. For the eight brucellosis patients with homologous MAT titers between 640 and 2,560, *F. tularensis* MAT titers were in the range 20–40 in seven patients, and 320 for the remaining patient. This study clearly showed that cross-reacting antibodies were absent (titer < 20) in the majority of tularemia (62.5%) and brucellosis (76.5%) patients. In most patients with cross-reacting antibodies, MAT titers to the heterologous antigen were lower than the cutoff. Finally, only seven tularemia and one brucellosis patients displayed cross-reacting antibody titers ≥160 to the heterologous antigen and thus could have led to diagnosis misinterpretation.

Bevanger et al. (1988) developed MAT tests using formalin-killed antigens from *F. tularensis*, *B. abortus*, or *Y. enterocolitica* O:3. They tested the first serum sample collected from 44 tularemia patients. Tularemia MAT titers were ≥80 for 40 (90.9%) samples, and between 20 and 40 for four samples. Sera from nine and two patients with tularemia showed cross agglutination with *B. abortus* and *Y. enterocolitica* O:3, respectively. However, heterologous antibody titers were much lower than homologous titers. Cross-reactions were abolished by dithiothreitol treatment of sera, suggesting IgM type antibodies were involved.

Sato et al. (1990) tested 50 serum samples from tularemia patients with two MAT tests using the Ootake strain of *F. tularensis* or the Nagashima strain of *B. abortus*. MAT titers against *F. tularensis* ranged from 10 to ≥10,240. All serum samples with *F. tularensis* MAT titers lower than 1,280 did not react with *B. abortus* antigen. Six of 15 samples with *F. tularensis* MAT titers higher than 2,560 displayed low-level cross-reacting antibodies against *B. abortus* (10–80, *i.e.*, at least 128 times lower than MAT titers against *F. tularensis*). Therefore, only serum samples with very high anti-*F. tularensis* agglutinin titers displayed some reactivity against *B. abortus*.

Using a combination of a home-made ELISA and Western blot, and an LPS extract from the LVS strain as an antigen, Schmitt et al. (2005) found no cross-reaction when testing five tularemia hyperimmune sera against *S. enterica* 0:30, *Brucella* sp., *E. coli* O:116 and O:157, *Y. enterocolitica* O:9, and *S. maltophilia*.

Splettstoesser et al. (2010) tested 58 sera from tularemia patients and 58 sera from patients infected with other bacterial pathogens using homemade ICT. No cross-reactions were observed with *F. novicida*, *F. philomiragia*, *Yersinia* *pestis*, *Burkholderia cepacia*, *B. mallei*, *B. pseudomallei*, and *Pseudomonas aeruginosa*. In contrast, Kiliç et al. (Kiliç et al., 2012) reported cross-reactions with tularemia serum samples against *Brucella* antigen when using the VIRapid ^®^ ICT.

Rastawicki et al. (2015) reported that 76/77 (98.7%) sera from tularemia patients gave a positive latex agglutination test. In contrast, this test was negative for sera from patients infected with *Yersinia* spp. (n = 86), *Salmonella* spp. (13), verotoxin-producing *E. coli* (8), *Borrelia burgdorferi* (11), *Legionella pneumophila* (11), and *Campylobacter jejuni* (10).

Serological cross-reactions have been reported for agglutination, indirect immunofluorescence, ICT, ELISA and Western blot techniques (Francis and Evans, 1926; Ransmeier and Ewing, 1941; Saslaw and Carlisle, 1961; Grunow et al., 2000; Schmitt et al., 2005; Sjöstedt, 2007; Chaignat et al., 2014). The main antigenic cross-reactions were observed between *F. tularensis* and both *Brucella* species and *Yersinia enterocolitica* serotypes O:3 and O:9. Lower level cross-reacting antibodies were reported with *Salmonella enterica* O:30, *Escherichia coli* 0:116 and O:157, and *Stenotrophomonas maltophilia* (Francis and Evans, 1926; Carlsson et al., 1979; Koskela and Herva, 1982b; Bevanger et al., 1988; Sato et al., 1990; Chaignat et al., 2014). These cross-reacting antibodies have been found in *F. tularensis* naturally infected and vaccinated persons (Saslaw and Carlisle, 1961). More recently, cross-reactions between *F. tularensis* and *Bartonella quintana* were reported, but this was likely related to the specific commercial IFA test (Petersson and Athlin, 2017).

Most studies have evaluated the reactivity of serum samples from tularemia patients against cross-reacting antigens (Carlsson et al., 1979; Bevanger et al., 1988; Sato et al., 1990; Gutiérrez et al., 2003; Schmitt et al., 2005). In this case, heterologous antibody titers were much lower than homologous titers. Fewer studies have evaluated the reactivity of sera from patients infected with cross-reacting pathogens against *F. tularensis* antigen (Behan and Klein, 1982; Rastawicki et al., 2015). Generally, no anti-*F. tularensis* antibodies could be detected in sera from these patients. Altogether, cross-reactions between *F. tularensis* and other human pathogens are usually weak and thus have little impact on routine serological diagnosis of tularemia. It should also be stressed that the clinical presentation and epidemiological context of the above mentioned infectious diseases are quite different.

## Seroepidemiological Studies On Tularemia

Koskela and Herva (1982b) evaluated the tularemia seroprevalence in Northern Finland. Sera from 1072 healthy adult blood donors collected in 12 rural communities of the Oulu province were tested by TAT. Sera from 168 blood donors had TAT titers ≥10. However, only the seven samples with TAT titers ≥80 were considered true positives, leading to a seroprevalence of 0.7%.

In 2001, Feldman (2003) performed a serosurvey in 132 professional landscapers working on Martha’s Vineyard (Massachusetts, USA), one year after a pneumonic tularemia outbreak had occurred (Feldman et al., 2001). Non-landscaper controls included 310 Martha’s Vineyard residents. The tularemia seroprevalence determined by MAT (cutoff ≥ 128) was significantly higher in landscapers (9.1%) than in controls (0.3%). Multivariate logistic regression analysis of data revealed the number of lawns mowed per week as the only significant risk factor. This result was correlated with the predominance of pulmonary forms of tularemia during the 2001 outbreak, suggesting air-borne transmission of *F. tularensis*. Landscapers infrequently used a mask during their outdoor activities.

In Canada, Lévesque et al. (1995) evaluated in 1992–1993 the tularemia seroprevalence (using a standard latex agglutination, cutoff ≥20) in 165 trappers living in the Quebec City area. Controls were people undergoing lipid testing, matched by age, sex, and area of residence. A tularemia seroprevalence of 2.4% was found in trappers compared to 0.6% in matched controls. The same authors (Lévesque et al., 2007) evaluated the tularemia seroprevalence in 50 people (22 men, 28 women) of the Cree community, a population living close to the wildlife fauna in the southern part of the Lake Mistassini. Only two men were found positive by TAT (cutoff ≥80) corresponding to a global seroprevalence of 4% but a 9.1% seroprevalence in men.

Following a large tularemia outbreak occurring in 1997–1998 in Castilla y Léon, Gutiérrez et al. (2003) tested sera collected between April 1996 and April 1997 from 4,825 people (51.6% women, 59.8% rural, and mean age 41.2 years) to check the presence of this disease in Spain before the outbreak. A positive MAT (titer ≥ 20) was found in nine individuals from four provinces (Burgos, Léon, Valladolid, and Zamora) corresponding to an overall seroprevalence of 0.19%. This study demonstrated that tularemia was present in Spain before the occurrence of the 1997–1998 outbreak.

In 2004, Porsch-Ozcürümez et al. (2004) evaluated the tularemia seroprevalence in 6,617 sera randomly collected in German people aged 18–79 years using a combination of homemade ELISA and western blot methods. Of 165 sera reaching the ELISA cutoff, only 15 were confirmed by western blot (*i.e.*, 0.23% seroprevalence).

Schmitt et al. (2005) used an ELISA test to evaluate the tularemia seroprevalence in 1,140 healthy young German adults. IgA, IgM and IgG specific antibodies were found in 10 (0.9%), 6 (0.5%) and 21 (1.8%) individuals, respectively. Because only positive ELISA tests confirmed by a homemade Western blot assay were considered true positives, the overall seroprevalence was 0.3%.

In 2006, Jenzora et al., (2008) evaluated the tularemia seroprevalence in 286 hunters randomly selected during a hunting exhibition in Dortmund (Germany). Positive ELISA and Western blot tests were found in five hunters (seroprevalence of 1.7%). In contrast, none of the 84 individuals hunting in the Berlin area was positive. The higher tularemia seroprevalence in the Dortmund group of hunters likely reflected a higher risk of exposure to *F. tularensis*-contaminated game animals and their arthropods.

Splettstoesser et al. (2009) evaluated the tularemia seroprevalence in 2416 people living in the urban area of Leutkirch (Baden-Wurttemberg, Germany). Using ELISA and Western blot techniques, specific antibodies were found in 56 (2.32%) tested persons. The seroprevalence was similar in men and women (1.07 *versus* 1.24%), although tularemia cases predominated in men. Higher seroprevalences were found in hunters (6.25%), farmers (3.94%), and people living for less than five years in Leutkirch (4.3–7.9%).

A tularemia serosurvey was performed in 2012 in northern Azerbaijan (Clark et al., 2012). Serum samples were collected in 2008 from 796 randomly selected people (347 men and 449 women; mean age, 36.2 years) living in 40 rural villages. Using a previously developed homemade ELISA (Chitadze et al., 2009), a high seroprevalence of 15.5% was found. This high seroprevalence contrasted with an infrequent report of symptoms that could correspond to tularemia. The seroprevalence was similar between men (13.8%) and women (16.8%) but was the highest in people aged 50 to 64 years (25%) and in the Xachmae region (17.2%).

In 2015, Zákutná et al. (2015) determined the seroprevalence of tularemia in 124 healthy blood donors (62.1% men, 66.1% urban) from Eastern Slovakia. Only nine (7.3%) persons were hunters and 12 (9.7%) exposed to agricultural fields. ELISA found a seroprevalence of 4% (five positive sera), but specific antibodies were detected by Western blot in only one individual. Seropositivity was associated with exposure to hay, straw, manure, or agricultural fields. Persons working in the agricultural sector were ten times more likely to be seropositive compared with those working in other sectors.

Tobudic et al. (2014) determined the tularemia seroprevalence in 546 Austrian individuals, including 226 professional soldiers and 320 civilians. Almost all participants were men (534, 97.8%), aged 18 to 60 years (median, 26 years). Specific IgM and IgG antibodies were detected by Serion ^®^ ELISA in three individuals, giving a seroprevalence of 0.5%. Interestingly, none of the tested soldiers had a positive tularemia serology, including those having in the past been on a mission abroad.

Rossow et al. (2015) determined the tularemia seroprevalence in Finland, a country with a high incidence rate of human tularemia. They tested 1045 serum samples randomly collected in all districts of Finland during a 2000–2001 national survey. Involved persons included 46% men and 54% women, of mean age 53 years (range 30–92 years). Positive sera were those with both a positive homemade ELISA (Koskela and Salminen, 1985) and Western blot. Sixteen sera were positive, giving a seroprevalence of 1.5%. No risk factor for tularemia seropositivity was found.

Büyük et al. (2016) evaluated the tularemia seroprevalence in 201 persons with occupational exposure to animals (103 farmers, 45 veterinarians, 42 butchers, and 11 hunters) in northern Turkey (Kars region, Anatolia). Specific antibody titers were determined using a homemade MAT and the Serazym ^®^ ELISA. Both tests were positive in 15 (7.5%) individuals (12 men and three women), including 14 (13.6%) farmers and one (2.2%) veterinarian. Seropositivity was not related to the length of professional experience. Farmers were found to be a risk group for tularemia, while human infections in Turkey are most often related to the consumption of *F. tularensis*-contaminated water.

Gazi et al. (2016) studied the tularemia seroprevalence in the Manisa province (western Aegean region of Turkey). In 2012, 450 sera were randomly collected in rural residents (48.1% men, 51.9% women, mean age of 49.2 years) of seven villages. Using the Serion ^®^ ELISA, a 7.1% seroprevalence was found (9.5% in women, 4.5% in men), correlating with the high tularemia endemic status of Turkey.

De Keukeleire et al. (2017) evaluated the tularemia seroprevalence in southern Belgium. Sera were collected in 2011 in three groups of people: 148 workers at risk of zoonoses, including 105 veterinarians, 34 farmers and nine hunters (86.5% men, mean age 49 years); 209 blood donors either living in rural areas (n = 209) or urban areas (193). Using the Serion ^®^ ELISA IgG, the seroprevalence was fourfold higher (2%) in exposed workers (especially veterinarians and farmers) than in the general rural and urban populations (0.5% each).

Recently, Njeru et al. (2017) evaluated the tularemia seroprevalence in 730 febrile patients (335 men and 395 women, 652 adults and 78 children) hospitalized in Garissa and Wajir hospitals (northeastern Kenya) in 2014–2015. The Serazym ^®^ ELISA tests detected 71 (9.7%) positive samples, of which only 27 (3.7%) were confirmed by Western blot. No risk factor for a positive *F. tularensis* serology was detected. This study suggests that tularemia may be present in Kenya, while the whole African continent is currently considered free of this disease. A single putative case of *F. tularensis* bacteremia has been reported recently in Sudan (Mohamed et al., 2012).

Akhvlediani et al. (2018) tested sera were collected in 500 military personnel (476 men) during routine examination in military clinics (October 2014 to February 2016) and in 697 people (310 men, 387 women) living in rural areas endemic for tularemia (October 2013 to September 2016). Ten samples were positive by MAT (cutoff ≥ 128) in the military personnel giving a 2% seroprevalence. Thirty-five samples were positive in people living in endemic areas (5.02% seroprevalence; 6.45% in men and 3.88% in women). Seropositive cases were more frequently in contact with several animal species.

Following a tularemia epizootic occurring in 2015 in the Devils Tower National Monument Park (Wyoming, USA), Harrist et al. (2019) determined the seroprevalence for this disease in the park employees. Among 44 persons working in the park, 23 (13 men) aged 21 to 40 years were tested by MAT. Titers ≥128 were found in three workers (13%), of which only one was diagnosed with tularemia. Primary risk factors for seropositivity were collecting animal carcasses and using a power blower in the park, and recreational hunting of prairie dogs in or outside the park.

Several seroepidemiological studies have been recently conducted in Iran (Esmaeili et al., 2014a; Esmaeili et al., 2014b; Esmaeili et al., 2019) using the Serion ^®^ ELISA test. A first study conducted in 2011–2012 in the Kurdistan province (Sanandaj, Marivan, and Sarvabad counties) was focused on hunters, butchers, and slaughterhouse workers (Esmaeili et al., 2014b). Serum samples were collected from 250 individuals (205 men and 45 women, mean age of 40.1 years). Contact with domestic animals was reported in 42% of persons and hunting in 32%. Positive ELISA tests were found in 36 (14.4%) samples, 14.1% in men versus 15.9% in women, and 16.1% in rural residents versus 7.8% in urban residents. The highest seroprevalences were found in hunters (18%), persons exposed to foxes (25%), and people over 51 years old (26.9%). A second study performed in 2011 in the Sistan and Baluchestan province (Esmaeili et al., 2014a) included 120 butchers and 64 slaughterhouse workers (all men, median age of 34 years). A positive ELISA was found in 12 (6.5%) individuals, including 6 (5%) butchers and 6 (9.4%) slaughterhouse workers. In 2015, a survey was conducted in the Ilam province (Esmaeili et al., 2019) in 112 ranchers, 79 farmers, 61 butchers and slaughterhouse workers, 34 Nature Conservation officers, 74 diagnostic laboratory referents. The participants were mostly men (76.3%), living in urban (158) or rural (159) areas, or nomadic (39). Ten of the 360 serum samples evaluated were detected positive by ELISA, giving a global seroprevalence of 2.8%. Higher seroprevalences were found in farmers (7.6%) and people aged 31 to 40 years (5.15%). No specific risk factor was found. In Iran, the first human case of tularemia was reported in 1980 (Esmaeili et al., 2014a; Esmaeili et al., 2014b; Esmaeili et al., 2019). Altogether, the above seroepidemiological studies strongly suggest that tularemia is still endemic in Iran.

Tularemia seroepidemiological studies are summarized in **Table 4**. These studies have evaluated the tularemia seroprevalence in specific areas, either in the general population or in specific groups of individuals. ELISA tests were performed in most recent studies because they allow easy testing of a large number of serum samples (Jenzora et al., 2008; Splettstoesser et al., 2009; Zákutná et al., 2015; Büyük et al., 2016; Njeru et al., 2017; Esmaeili et al., 2019). Overall, most studies have reported low seroprevalences (0.19 to 4%) in the general populations studied (Koskela and Herva, 1982b; Gutiérrez et al., 2003; Porsch-Ozcürümez et al., 2004; Schmitt et al., 2005; Jenzora et al., 2008; Splettstoesser et al., 2009; Tobudic et al., 2014; Rossow et al., 2015; Zákutná et al., 2015; De Keukeleire et al., 2017; Njeru et al., 2017; Akhvlediani et al., 2018). Higher seroprevalences (5.02 to 18%) have been reported in tularemia highly endemic areas, in rural populations, in hunters, and in specific occupational groups (including farmers, butchers, slaughterhouse workers, ranchers, and park workers) (Feldman, 2003; Splettstoesser et al., 2009; Clark et al., 2012; Esmaeili et al., 2014a; Esmaeili et al., 2014b; Büyük et al., 2016; Gazi et al., 2016; Akhvlediani et al., 2018; Esmaeili et al., 2019; Harrist et al., 2019).

**Table 4:** Reported tularemia seroprevalence in the general population or specific at-risk groups.

Reference	Country	Year of serum sampling*	Population	Serological test (cutoff titer)	Seroprevalence
(Koskela and Herva, 1982b)	Finland (Oulu province)		1,072 healthy blood donors	TAT (≥80)	0.7%
(Lévesque et al., 1995)	Canada (Quebec City area)	1992**–**1993	165 trappers (95.2% men, mean age 40 years)	LAT (≥20)	2.4% (controls, 0.6%)
(Feldman et al., 2003)	USA (Martha’s Vineyard, Massachusetts)	2001	132 landscapers	MAT (≥128)	9.1%
			310 healthy residents		0.3%
(Gutiérrez et al., 2003)	Spain (Castilla y Léon region)	1996**–**1997	4,825 people (48.4% men, mean age 41.2 years, 59.8% rural)	MAT (≥20)	0.19%
(Porsch-Ozcürümez et al., 2004)	Germany		6,617 people aged 18**–**79 years	ELISA (NA) and western blot	0.23%
(Schmitt et al., 2005)	Germany		1,140 healthy adults	ELISA (NA)	0.3%
(Lévesque et al., 2007)	Canada (Lake Mistassini, Quebec province)		50 Cree community people (22 men, 28 women)	TAT (≥80)	4% (men, 9.1%, no woman)
(Jenzora et al., 2008)	Germany		286 hunters	ELISA (NA) and western blot	1.7%
(Splettstoesser et al., 2009)	Germany (Leutkirch, (Baden-Wurttemberg)		2,416 people living in urban areas	ELISA (NA) and western blot	2.32% (hunters, 6.25%; farmers, 3.94%)
(Clark et al., 2012)	Azerbaijan	2008	796 people (43.6% men; mean age, 36.2 years; 100% rural)	ELISA (NA)	15.5% (50**–**64 years, 25%)
(Zákutná et al., 2015)	Slovakia		124 healthy blood donors (62.1% men; 66.1% urban)	ELISA (NA) and western blot	4%
(Tobudic et al., 2014)	Austria		546 people (226 soldiers and 320 civilians; 97.8% men; median age, 26 years)	Serion ^®^ ELISA	civilians, 0.7% soldiers, 0%
(Rossow et al., 2015)	Finland	2000**–**2001	1,045 people (national survey; 46% men; mean age, 53 years)	ELISA (NA) and western blot	2%
(Büyük et al., 2016)	Turkey (Kars region, Eastern Anatolia)		201 people (88.6% men; 103 farmers, 45 veterinarians, 42 butchers, and 11 hunters)	MAT and Serazym ^®^ ELISA	7.5% (farmers, 13.6%; veterinarians, 2.2%)
(Gazi et al., 2016)	Turkey (Manisa province, Aegean region)	2012	450 people (48.1% men; mean age, 49.2 years; rural)	Serion ^®^ ELISA	7.1% (women, 9.5%; men, 4.5%)
(De Keukeleire et al., 2017)	Belgium (Wallonia)	2011	148 people at risk for zoonoses (105 veterinarians, 34 farmers, and nine hunters; 86.5% men; mean age 49 years)	Serion ^®^ ELISA	2%
			209 blood donors living in rural areas (56.7% men, mean age 43 years)		0.5%
			193 blood donors living in urban areas		0.5%
(Njeru et al., 2017)	Kenya (northeastern, Garissa and Wajir hospitals)	2014**–**2015	730 febrile hospitalized patients (45.9% men; 652 adults and 78 children)	Serazym ^®^ ELISA and western blot	3.7%
(Akhvlediani et al., 2018)	Georgia	2014**–**2016	500 healthy military personnel (95.2% men)	MAT (≥128)	2%
		2013**–**2016	697 people (44.5% men; rural areas)		5.02% (men, 6.45%; women, 3.88%)
(Harrist et al., 2019)	USA (Devils Tower National Monument park, Wyoming, USA)	2015	23 persons working in the park (13 men, aged 21 to 40 years)	MAT (≥128)	13%
(Esmaeili et al., 2014b)	Iran (Kurdistan province)	2011**–**2012	250 people (82% men; mean age, 40.1 years; contact with domestic animals, 42%; hunting, 32%)	Serion ^®^ ELISA	14.4% (hunters, 18%; >51 years, 26.9%; rural, 16.1%)
(Esmaeili et al., 2014a)	Iran (Sistan and Baluchestan province)	2011	184 people (100% men; 120 butchers and 64 slaughterhouse workers; median age, 34 years).	Serion ^®^ ELISA	6.5% (butchers, 5%; slaughterhouse workers, 9.4%)
(Esmaeili et al., 2019)	Iran (Ilam province)	2015	360 people (76.3% men; rural, 44.7%; 79 farmers, 61 butchers, and slaughterhouse workers)	Serion ^®^ ELISA	2.8% (Farmers, 7.6%; butchers, 3.3%; rural, 3.8%)

*. The year of serum sampling is not specified in many studies. We considered that, in most cases, it was close to the year of the corresponding publication. TAT, tube agglutination test; MAT, microagglutination tests; LAT, latex agglutination test; IFA, immunofluorescence assay; ELISA, enzyme-linked immunosorbent assay; ICT, immunochromatographic assay.

As for many bacterial diseases, serological methods have limitations that reduce the reliability and public health significance of the determined seroprevalences. Previously mentioned cross-reactions between *F. tularensis* and other microorganisms might lead to false-positive results and thus overestimation of the tularemia seroprevalence. Cutoff titers used to define a positive serology are usually much lower for seroepidemiological studies than for tularemia diagnosis. As indicated before, the risk of false-positive results due to antigenic cross-reactions increases when considering low antibody titers (Syrjälä et al., 1986). In some studies, authors confirmed positive serological tests using Western blot to reduce false-positive results caused by antigenic cross-reactions (Jenzora et al., 2008; Splettstoesser et al., 2009; Zákutná et al., 2015; Njeru et al., 2017). Besides, in many instances, the Western blot did not confirm positive ELISA tests, especially when ELISA titers were just above the cutoffs (Schmitt et al., 2005; Rossow et al., 2015). This discrepancy may reflect the higher specificity but also, the lower sensitivity of the Western blot technique, with potential underevaluation of the tularemia seroprevalence. Another limitation of tularemia seroprevalence studies is related to the long-term persistence of specific antibodies after *F. tularensis* infection. This phenomenon increases the seroprevalence, which may not correlate with the observed prevalence of human tularemia cases in a specific area or population. Hence, some studies have highlighted the low number of reported tularemia cases despite a high seroprevalence in a given population (Clark et al., 2012; Esmaeili et al., 2014a; Esmaeili et al., 2014b; Njeru et al., 2017; Esmaeili et al., 2019). Other explanations may include under-diagnosis and under-reporting of the disease. The Western blot gives similar patterns with recently developed or residual antibodies (Bevanger et al., 1994). Despite these well-known limitations, when using the same methodology, significant variations in the tularemia seroprevalence over time in specific geographic areas or populations may reveal variations in disease epidemiology.

## Current Limitations Of Tularemia Serology

Current limitations in the methodology and evaluation of tularemia serological methods can be highlighted considering the recent STARD guidelines (Bossuyt et al., 2015; Cohen et al., 2016).

### Criteria and Identification of the Eligible Participants of Studies

#### Tularemia Case Definition

There is no current gold standard for tularemia diagnosis. The clinical and epidemiological findings in tularemia patients are often poorly specific (Sjöstedt, 2007; Maurin and Gyuranecz, 2016). The overall sensitivity of *F. tularensis* culture is low (usually <10%), and PCR-based techniques are only useful for specific situations (*e.g.*, testing lymph node aspirates or tissues when available) (Tärnvik and Chu, 2007; Hepburn and Simpson, 2008). Serological tests are considered the most sensitive diagnostic tests. However, they have also many limitations: a low sensitivity during the first two weeks of disease evolution; no significant antibody response in a few patients; and more importantly false-positive results due to serological cross-reactions or residual antibodies in patients with past *F. tularensis* infection (Schmitt et al., 2005; Tärnvik and Chu, 2007; Hepburn and Simpson, 2008; Chaignat et al., 2014; Maurin and Gyuranecz, 2016). Therefore, an accurate definition of tularemia patients must combine several clinical, epidemiological, and microbiological criteria. Compatible clinical findings include all signs and symptoms that are usually found in tularemia patients, even if poorly specific. Compatible epidemiological findings are at least residence or travel in known tularemia endemic areas. Risk factors such as contact with animals (mostly game animals and rodents), arthropod bites (ticks, and mosquitoes in Sweden and Finland), and exposure to other well-known infection sources have to be considered, although this information is often missing. As for microbiological criteria, using a single positive serological test (especially a positive MAT) to define tularemia cases is not acceptable for the evaluation of the clinical performances of a tularemia diagnostic test. Tularemia diagnosis should be considered as confirmed when: 1/*F. tularensis* has been isolated from any clinical sample; 2/*F. tularensis* DNA has been detected in any clinical sample; or 3/a seroconversion or fourfold or higher rise in antibody titers has been observed with any serological tests. Although imperfect, this case definition allows including tularemia patients, whatever the stage of the disease. Ideally, the evaluated patients’ group should include independent cases of various clinical forms and at various stages of the disease. This situation will allow the evaluation of diagnostic tests in a real clinical situation, and thus results would be more comfortable to extrapolate to other geographic areas.

#### Control Patients

The control group should classically include healthy persons, patients involved with other infectious diseases (especially diseases with symptoms similar to those of tularemia), and patients involved with diseases well known to induce false-positive results (*e.g.*, Infectious mononucleosis caused by Epstein Bar virus). Healthy blood donors alone do not constitute an acceptable control group. Patients tested for tularemia because of compatible clinical findings, but for which recent *F. tularensis* infection has been eliminated with certainty constitute a useful control group. Lack of false-positive results for the evaluated test in the control group is highly suspicious because of known antigenic cross-reactions between *F. tularensis* and other pathogens and long-term persistence of specific antibodies in patients with past tularemia infection. Finding a 100% specificity (no false positive in control patients) for a newly evaluated serological test for tularemia often reflects poor relevance or insufficient size of the control group.

### Consecutive, Random or Convenience Series

Consecutive tularemia cases are often included in the patients’ group. It is an easy way to avoid bias selection, although the results of the evaluation of a tularemia diagnostic test may vary according to the period of serum sample collection. Indeed, the results of a diagnostic test may not be the same in two different groups of patients, especially according to the sex ratio, mean age, predominant clinical manifestations, modes of infections, etc. Random selection of patients may be difficult for tularemia because the disease is rare, and patient cohorts are usually limited. Convenience series are often used, *e.g.*, patients diagnosed during a tularemia outbreak. In this situation, there is usually a predominant mode of infection and clinical presentation of the disease. Besides, the infected population may have specific characteristics. Although of interest, this kind of study may be more difficult to extrapolate to other clinical and epidemiological situations (see section *Demographic and Relatedness of Tularemia Cases*).

### Choice of the Reference Standard Test and Its Execution

Although the MAT is still considered a reference diagnostic test for tularemia by the WHO (World Health Organization, 2007), this technique is not 100% sensitive and specific. Moreover, the MAT methodology developed in different diagnostic laboratories is heterogeneous, especially in terms of the antigen used (*F. tularensis strain* and antigen preparation). The combination of MAT with a more specific test such as immunoblot does not solve the problem of false-positive results caused by residual antibodies of past infection (Bevanger et al., 1994). Many studies have compared the performances of a new serological test to the classical MAT for tularemia diagnosis (Kiliç et al., 2012). This strategy can at best evaluate the concordance of results between the new assay and the MAT. However, the performances of the evaluated test will be, at best, equal or inferior to those of the MAT. In a real clinical situation, because the MAT is not a correct “gold standard,” interpretation of “false positive” or “false negative” results according to MAT may be erroneous. Tularemia cases not diagnosed by the MAT may be real infections detected by the newly evaluated assay. This situation is especially true for ELISA tests, which are more sensitive than the MAT during the first two weeks following disease onset (Viljanen et al., 1983; Syrjälä et al., 1986; Yanes et al., 2018). Besides, in the literature, many culture-positive tularemia cases were not confirmed by serology (Helvaci et al., 2000). Thus, using the MAT as a reference obviously can lead to erroneous evaluation of the sensitivity and specificity of the evaluated test. Currently, none of the available serological tests can be used to define tularemia and non-tularemia cases. A more accurate evaluation of the clinical usefulness of a newly developed serological test requires an *a-priori* definition of a tularemia case and as indicated above, should include a combination of clinical, epidemiological, and microbiological criteria.

### Demographic and Relatedness of Tularemia Cases

The performances of serological tests may not be the same when evaluating a group of patients with different sex ratio or mean age, because antibody response may vary according to the infected hosts investigated. This limitation is also valid when evaluating patients infected through similar exposure, during a short period, and possibly by a single *F. tularensis* strain (*e.g.*, during a tularemia outbreak) compared to the situation of sporadic independent cases, which is the most frequent situation for tularemia. In the first situation, patients are more likely to have a similar clinical presentation and a more homogeneous immune response. Also, after the outbreak situation has been recognized, clinicians may more readily and accurately recognize tularemia cases. Thus, the delay between the onset of symptoms and serological testing should be reduced and less variable in the event of an epidemic. The diagnostic sensitivity of serological tests is, therefore, likely to be higher in an epidemic situation than when testing sporadic unrelated cases. More generally, many studies in the literature have been focused on small groups of tularemia cases occurring on a limited time scale. This situation also raises the question of the potential link between the studied tularemia cases, the number and the genetic relatedness of the *F. tularensis* strains involved, and, ultimately, the representativeness of the patient population studied compared to the general population potentially exposed to this pathogen.

### Disease Evolution and Severity

Patients suffering from different clinical forms of tularemia may consult after a few days to several weeks following disease onset. Patients with acute diseases (*e.g.*, acute pneumonia or severe typhoidal forms) or with severe pain (*e.g.*, the oropharyngeal and oculoglandular forms) usually seek medical attention very early after infection, a time when serological tests will be most often negative. In contrast, many patients suffering from glandular or ulceroglandular forms of tularemia will develop mild symptoms over several weeks, and consult their general practitioner at a time when serological tests are most often positive. Besides, tularemia clinical suspicion may vary according to the specificity of clinical manifestations, which changes the accuracy of a diagnostic test. An ulceroglandular form of tularemia is more straightforward to diagnose than a pneumonic or typhoidal form. Severe diseases are more likely to be diagnosed, mainly because a large number of investigations will be performed in a short period. Thus, although the overall sensitivity of serological tests has been reported to be similar among the different clinical forms of tularemia (Syrjälä et al., 1986), the practical situation is that the likelihood that the significant antibody titers will be detected (especially in the first collected serum sample) greatly varies according to the clinical presentation and the severity of disease. Therefore, the patients’ group should be as possible representative of the general population of tularemia cases in a specific geographic area. Studies performed in different countries where the predominant clinical form of tularemia is not the same, or severity of the disease is different (*e.g.*, type A *versus* type B infections) may not give similar clinical sensitivity and specificity results for the newly evaluated but also the comparative serological tests.

### Disease Prevalence

It is well known that the positive (PPV) and negative (NPV) predictive values of a serological test will significantly vary according to the prevalence of the disease in the studied area, and high variations in tularemia prevalence exist between different endemic areas. PPV and NPV can only be reliably assessed if the study population is representative of the general population, which is often difficult to achieve and depends on the epidemiological situation where the test is used. Homemade and commercial tests for tularemia diagnosis are usually evaluated in the country’s population in which the test is intended to be used. Both the patient and control groups must be made up of people belonging to this same population. These tests may not perform as well in other populations, especially if the epidemiological situation is quite different.

### Feasibility of Serological Tests and Generalizability of Results

Serological tests allow diagnostic confirmation of tularemia in case of seroconversion or fourfold or higher rise in antibody titers between two sera taken at least two weeks apart (acute and convalescent-phase sera). A significant limitation is that few patients have two blood samples collected on a routine basis. Only one blood sample is usually collected at the time of the first medical consultation, thus, after a variable time of evolution of the disease. As an example, seroconversion was demonstrated in only 19 (13.4%) of the 142 patients suffering from tularemia during the large outbreak that occurred in Spain in 1997-1998 (Pérez-Castrillón et al., 2001). Therefore, the diagnostic sensitivity of tularemia serological tests dramatically depends on the patient population studied, the mean delay in the clinical suspicion of tularemia and serological testing, and the availability of pairs of serum samples.

Another limitation is the lack of standardization of serological tests for tularemia. The *F. tularensis* strain used and the method of antigen preparation greatly vary between different diagnostic laboratories. When evaluating the performances of these tests, a lack of standardization makes difficult the comparison of results between different diagnostic laboratories. Commercial serological tests are intended to be used worldwide. However, these tests are rarely validated in different countries with different epidemiological situations, including a low or high prevalence of the disease. Notably, it would be expected that cutoff titers are different according to the populations in which these tests are used. These recommendations can be considered general regarding infectious diseases, but are particularly relevant for zoonoses with a specific distribution such as tularemia.

### Intended Use and Clinical Role of Serological Tests

The evaluation of a new serological test for tularemia should lead to recommendations on the clinical use of the test. Showing that a new test is as sensitive and specific as the reference MAT test is not sufficient to define its medical interest and its mode of use in routine diagnostic practice. The test could be used as a screening test (very high sensitivity) or as a confirmatory test (very high specificity). The test sensitivity and specificity should also be evaluated according to the time of evolution and severity of the disease. The type and frequency of false positive and false negative results and serological cross-reactions should be specified.

## Conclusion

Tularemia diagnosis remains primarily based on serological methods. However, tularemia serological tests are varied and poorly standardized. None of these tests can be currently considered as a reference diagnostic test. All have limitations in terms of sensitivity and specificity. Their combination (*e.g.*, MAT, IFA, or ELISA with immunoblot) may increase the diagnostic specificity but usually lead to reduced sensitivity. The more recently developed ELISA tests are highly sensitive and allow earlier diagnosis than other methods. However, they have also been associated with false-positive results. Most tularemia cases can be diagnosed according to compatible clinical and epidemiological findings and a positive serological test. However, the clinical spectrum and epidemiology of tularemia are evolving. As examples, the disease has recently been reported in patients treated with immunosuppressive drugs (Calin et al., 2017), and in patients with vascular or orthopedic prostheses (Briere et al., 2015; Rawal et al., 2017; Chrdle et al., 2019). Also, endemic areas of tularemia have been extended to the southern hemisphere, especially Australia, where possums represent a newly described reservoir of *F. tularensis* (Aravena-Román et al., 2015). Thus, more accurate tests are needed to confirm the diagnosis in patients with unusual or previously undescribed clinical manifestations. More importantly, serological tests should be standardized on a global scale to make a comparison of clinical and epidemiological data between countries more relevant.

## Author Contributions

The author confirms being the sole contributor of this work and has approved it for publication.

## Funding

This work was supported by the Direction Generale de l’Armement (DGA, ANR-17-ASTR-0024).

## Conflict of Interest

The author declares that the research was conducted in the absence of any commercial or financial relationships that could be construed as a potential conflict of interest.
